# Supplementary material for: Implementation of Microcirculation Examination in Clinical Practice—Insights from the Nationwide POL-MKW Registry
Source: Medicina (Kaunas). 2024 Feb 5;60(2):277. doi: 10.3390/medicina60020277 (PMC10890290; doi:10.3390/medicina60020277)
Supplement: Supplementary file 1 [file medicina-60-00277-s001.zip › medicina-2757212-supplementary.pdf]

Table S1. Demographic and clinical characteristics

Variable	Total<br>N = 223	CFR ≤2<br>N = 91	CFR >2<br>N = 117	*P*-value	IMR ≥25<br>N = 84	IMR <25<br>N = 121	*P*-value
Median age, years	66.2 [59.9; 71.9]	67.7 [60.5; 73.1]	65.5 [57.4; 70.6]	0.06	65.4 [58.4; 71.7]	66.8 [58.4; 71.7]	0.54
Gender, males	120 (55.0)	51 (56.0)	66 (56.9)	0.90	43 (51.2)	72 (60.0)	0.21
BMI, kg/m^2	28.0 [24.9; 31.2]	27.8 [24.8; 31.2]	28.3 [25.0; 32.1]	0.73	28.0 [25.5; 31.2]	27.9 [24.8; 31.9]	0.75
Obesity (BMI>30)	69 (33.3)	28 (32.2)	38 (33.9)	0.80	26 (31.7)	39 (33.9)	0.75
Diabetes mellitus	76 (35.0)	34 (37.8)	39 (33.3)	0.51	34 (40.5)	38 (31.7)	0.20
Prior cerebral stroke	22 (10.1)	11 (12.1)	11 (9.4)	0.53	13 (15.5)	9 (7.4)	0.07
Prior MI	70 (32.3)	32 (35.6)	36 (30.8)	0.47	16 (19.3)	51 (42.1)	<0.001
Prior PCI	70 (32.7)	27 (29.7)	41 (35.7)	0.37	15 (17.9)	52 (43.7)	<0.001
Prior CABG	6 (2.8)	1 (1.1)	5 (4.3)	0.17	1 (1.2)	5 (4.2)	0.21
Smoking history	116 (54.0)	57 (62.6)	54 (46.2)	0.02	44 (52.3)	65 (53.7)	0.85
Current smoker	38 (17.7)	17 (18.7)	21 (17.9)	0.89	10 (11.9)	28 (23.1)	0.04

Arterial hypertension	187 (85.8)	79 (86.8)	99 (84.6)	0.65	69 (82.1)	107 (88.4)	0.20
Kidney disease	26 (11.9)	15 (16.5)	10 (8.5)	0.51	11 (13.1)	14 (11.6)	0.74
Family history of CVD	55 (26.8)	25 (28.4)	29 (25.9)	0.69	23 (27.7)	30 (26.3)	0.83
IHD	160 (75.5)	62 (68.9)	92 (80.0)	0.07	45 (53.6)	107 (89.9)	<0.001
COPD	15 (6.9)	8 (8.9)	7 (6.0)	0.42	5 (6.0)	9 (7.5)	0.67
Asthma	10 (4.6)	3 (3.3)	7 (6.0)	0.29	3 (3.6)	7 (5.8)	0.35
PAD	21 (9.9)	9 (9.9)	11 (9.6)	0.95	6 (7.1)	14 (11.9)	0.27
PE/DVT	15 (7.1)	11 (12.2)	4 (3.5)	0.02	11 (13.1)	4 (3.4)	0.01
Thyroid disease				0.28			0.04
- Hyperthyroidsm	5 (2.3)	1 (1.1)	4 (3.4)		0 (0.0)	5 (4.1)	
- Hypothyroidsm	27 (12.4)	14 (15.4)	11 (9.4)		8 (9.5)	17 (14.0)	
- Other	6 (2.8)	1 (1.1)	4 (3.4)		0 (0.0)	5 (4.1)	
AF	42 (19.4)	23 (25.3)	19 (16.2)	0.11	23 (27.4)	18 (14.9)	0.03
Hyperlipidaemia		67 (73.6)	85 (73.9)	0.96	57 (68.7)	93 (77.5)	0.16
Prior PTA/CAS/other	13 (6.1)	7 (7.7)	5 (4.4)	0.32	6 (7.1)	6 (5.1)	0.54

Days of hospitalization	3 [2; 5]	3 [3; 5]	3 [2; 4]	0.15	3 [3; 4]	3 [2; 5]	0.66

Data are presented as median [Q1; Q3] and counts (percentages).

AF: atrial fibrillation; BMI: Body Mass Index; CABG: coronary artery by-pass grafting; CAS: carotid artery stenting; CFR: coronary flow reserve; COPD: chronic obstructive pulmonary disease; CVD: cardiovascular disease; DVT: deep venous thrombosis; IHD: ischaemic heart disease; IMR: index of microcirculatory resistance; MI: myocardial infarction; PAD: peripheral artery disease; PCI: percutaneous coronary intervention; PE: pulmonary embolism; PTA: percutaneous transluminal angioplasty

Table S2 Examination findings

Variable	Total<br>N = 223	CFR ≤2<br>N = 91	CFR >2<br>N = 117	*P*-value	IMR≥25<br>N = 84	IMR<25<br>N = 121	*P*-value
SBP	136.5 ± 19.9<br>135 [124; 147]	135.5 ± 19.8<br>135 [120; 145]	137.3 ± 20.4<br>135.5 [124; 147]	0.46	132.8 ± 18.4<br>130 [120.5; 143.5]	139.1 ± 21.0<br>137.5 [125; 150]	0.03

DBP	79.7 ± 11.5<br>80 [70; 88]	79.6 ± 12.2<br>80 [70; 88]	79.8 ± 11.3<br>80 [70; 86]	0.90	78.0 ± 11.8<br>80 [70; 85.5]	80.6 ± 11.5<br>80 [71; 89]	0.14
HR	71.9 ± 13.5<br>70 [62; 80]	73.5 ± 14.2<br>73 [62; 80]	70.9 ± 12.8<br>70 [61; 79.5]	0.13	71.7 ± 14.3<br>70 [60; 80]	72.1 ± 13.0<br>70 [62; 80]	0.72
SpO_2	96.1 ± 2.2<br>97 [95; 98]	96 ± 2.4<br>96.5 [95; 98]	96.1 ± 1.0<br>97 [95; 98]	0.95	95.7 ± 2.2<br>96 [94; 98]	96.4 ± 2.2<br>97 [96; 98]	0.03
IVSd, mm	11 [10; 12]	11 [10; 12]	11 [10; 12]	0.95	11 [10; 12]	11 [9; 12]	0.76
LVEF	55 [47; 60]	55 [40; 60]	56 [50; 60]	0.02	55 [50; 60]	55 [45; 60]	0.65
LVEDD, mm	49 [45; 53]	50 [45; 57]	49 [45; 53]	0.20	49,5 [45; 53]	50 [46; 54]	0.69
RVEDD, mm	32 [28; 35]	33 [30;39]	32 [28; 34]	0.04	33 [30; 36]	31 [28; 34]	0.02
LBBB	10 (5.0)	4 (4.7)	6 (5.4)	0.54	2 (2.5)	8 (7.0)	0.14
RBBB	8 (4.0)	5 (5.8)	3 (2.7)	0.23	4 (4.9)	4 (3.5)	0.44
AV II block	3 (1.5)	2 (2.3)	1 (0.9)	0.41	1 (1.2)	2 (1.8)	0.63
Leucocytes (10^3/µl)	7.4 [6.3; 9.0]	7.4 [6.39; 9.12]	7.5 [6.1; 8.9]	0.61	7.0 [6.0; 8.99]	7.70 [6.62; 9.03]	0.08
Haemoglobin (g/dl)	14.2 [13.1; 15.2]	14.3 [13.2; 15.2]	14.0 [13.0; 15.2]	0.48	14.2 [13.2; 15.4]	14.1 [13.0; 15.2]	0.42

Haematocrit (%)	41.2 [38.8; 43.5]	42.0 [39.0; 43.9]	41.0 [38.5; 43.5]	0.69	42 [39; 43.6]	41.0 [38.4; 43.5]	0.27
Erythrocytes (10^6/µl)	4.60 [4.30; 4.93]	4.62 [4.30; 4.91]	4.57 [4.28; 4.95]	0.96	4.62 [4.30; 4.95]	4.57 [4.27; 4.90]	0.47
Creatinine (umol/l)	83 [72; 102]	80.5 [70.0; 102.8]	84.0 [73.0; 99.0]	0.66	81.0 [70.0; 100.0]	83.94 [72.5; 102.8]	0.33
eGFR							
<30	1 (0.5)	1 (1.1)	0 (0.0)	0.31	1 (1.2)	0 (0.0)	0.55
30-60	39 (18.3)	18 (20.0)	1 (11.1)		13 (15.7)	24 (20.3)	
60-90	152 (71.4)	64 (71.1)	7 (77.8)		58 (69.9)	85 (72.0)	
>90	21 (9.9)	7 (7.8)	1 (11.1)		11 (13.3)	9 (7.6)	
NT-pro BNP (pg/ml)	230.5 [126.0; 746.0]	232 [131; 1,522]	235 [125; 668]	0.71	246 [125; 1,486]	193 [110; 668]	0.54
Hs Troponin T (ng/l)	14.0 [8.0; 49.0]	11.5 [6.0; 27.0]	17.0 [10.0; 60.0]	0.08	16.0 [7.5; 35.0]	13.5 [8.5; 51.0]	0.77
Hs Troponin I (ng/l)	4.0 [2.0; 9,9]	4.0 [2.0; 8.8]	4.3 [3.0; 11.0]	0.60	3.7 [2.0; 8.0]	5.5 [3.5; 314.5]	0.02
CK-Mb mass (ng/ml)	1.8 [1.0; 7.0]	2.2 [1.2; 5.0]	1.6 [1.0; 7.0]	1	1.6 [1.2; 10.0]	2.2 [1.0; 5.8]	1

CK-Mb activity (U/I)	29 [18; 106]	18.0 [16.0; 29.0]	138 [27; 166]	0.14	67.5 [29.0; 106.0]	22.5 [16.0; 138.0]	0.62
Coronary Noninvasive Diagnostic Testing	71 (31.8)	38 (41.8)	31 (26.5)	0.03	47 (56.0)	21 (17.4)	<0.001

Data are presented as median [Q1; Q3], mean ± standard deviation and counts (percentages).

CFR: coronary flow reserve; DBP: diastolic blood pressure; eGFR: estimated glomerular filtration rate; HR: heart rhythm; IMR: index of microcirculatory resistance; IVSd: interventricular septum diameter; NT-pro BNP: N-terminal pro b-type natriuretic peptide; LBBB: left bundle branch block; LVEDD: left ventricle end-diastolic diameter; LVEF: left ventricle ejection fraction; RBBB: right bundle branch block; RVEDD: right ventricle end-diastolic diameter; SBP: systolic blood pressure

Table S3. Pharmacotherapy

Variable	Total N = 223	CFR ≤2 N = 91	CFR >2 N = 117	*P*-value	IMR≥25 N = 84	IMR<25 N = 121	*P*-value
ASA	147 (68.7)	58 (65.9)	82 (70.7)	0.47	54 (65.1)	85 (72.0)	0.29
Statin	178 (82.0)	72 (80.0)	98 (83.8)	0.48	65 (78.3)	102 (84.3)	0.28

P_2Y12 inhibitors							
- Clopidogrel	38 (17.0)	17 (18.7)	21 (17.9)	0.28	9 (10.7)	29 (24.0)	0.002
- Ticagrelor	12 (5.4)	2 (2.2)	10 (8.5)		1 (1.2)	11 (9.1)	
- Prasugrel	2 (0.9)	1 (1.1)	1 (0.9)		0 (0.0)	2 (1.7)	
Antithrombotic treatment							
- NOAC	40 (17.9)	21 (23.1)	18 (15.4)	0.29	23 (27.4)	15 (12.4)	0.02
- OAC	5 (2.2)	3 (3.3)	2 (1.7)		3 (3.6)	2 (1.7)	
- LMWH	8 (3.6)	2 (2.2)	6 (5.1)		1 (1.2)	7 (5.8)	
- Other	2 (0.9)	0 (0.0)	2 (1.7)		0 (0.0)	2 (1.7)	
ACEI	134 (62.0)	56 (62.9)	72 (61.5)	0.84	46 (56.1)	81 (66.9)	0.12
BB	157 (73.0)	65 (73.0)	86 (74.1)	0.86	61 (73.5)	88 (73.9)	0.94
CCB	72 (33.5)	22 (25.0)	44 (37.6)	0.06	19 (23.2)	47 (39.2)	0.02
DIU	87 (40.5)	33 (37.1)	51 (44.0)	0.32	30 (36.1)	52 (43.7)	0.28
AB	14 (6.6)	3 (3.4)	10 (8.8)	0.12	5 (6.1)	7 (5.9)	0.59

PPI	81 (38.0)	34 (38.2)	43 (37.1)	0.87	32 (38.6)	43 (36.1)	0.73
Oral Hypoglycaemic Medications	67 (31.0)	32 (36.0)	31 (26.5)	0.14	28 (33.7)	34 (28.3)	0.41
Insulin	17 (8.0)	4 (4.5)	13 (11.3)	0.07	4 (4.9)	13 (10.9)	0.10
Fibrates	8 (3.7)	3 (3.4)	5 (4.3)	0.52	1 (1.2)	7 (5.8)	0.09

Data are presented as counts (percentages).

AB: alfa blockers; ACEI: angiotensin-converting enzyme inhibitors; ASA: acetylsalicylic acid; BB: beta blockers; CCB: calcium channel blockers; CFR: coronary flow reserve; DIU: diuretics; IMR: index of microcirculatory resistance; NOAC: novel oral anticoagulant; PPI: proton pump inhibitors; OAC: oral anticoagulant; LMWH: low molecular weight heparin

Table S4. Coronary microcirculatory assessment

Variable	Total<br>N = 223	CFR ≤2<br>N = 91	CFR >2<br>N = 117	*P*-value	IMR≥25<br>N = 84	IMR<25<br>N = 121	*P*-value
RFR	0.92 [0.88; 0.95]	0.92 [0.87; 0.95]	0.92 [0.89; 0.94]	0.82	0.94 [0.91; 0.96]	0.91 [0.86; 0.93]	<0.001
FFR	0.88 [0.83; 0.92]	0.90 [0.83; 0.92]	0.88 [0.83; 0.92]	0.29	0.9 [0.95; 0.93]	0.87 [0.79; 0.92]	0.006
High FFR (>0.8)	202 (81.1)	75 (81.5)	107 (79.9)	0.76	81 (95.4)	97 (71.3)	<0.001
Impaired FFR (≤0.8)	47 (18.9)	17 (18.5)	27 (20.2)	0.76	4 (4.6)	39 (28.7)	<0.001
CFR	2.3 [1.6; 3.4]	1.4 [1.0; 1.75]	3.1 [2.5; 4.2]	<0.001	2.0 [1.3; 2.5]	2.9 [1.9; 4.0]	<0.001
IMR	20.0 [13.0; 33.0]	28.0 [16.0; 44.0]	17.0 [12.0; 26.0]	<0.001	37 [29; 49]	14 [11; 19]	<0.001
Assessed artery							
None	17 (6.8)	-	-		-	-	
LAD	183 (73.5)	75 (81.5)	108 (80.6)	0.94	74 (85.1)	106 (77.9)	0.73
Cx	25 (10.0)	10 (10.9)	12 (9.0)		8 (9.2)	13 (9.6)	
Mg	4 (1.6)	2 (2.2)	2 (1.5)		1 (1.1)	3 (2.2)	

Dg<br>RCA	6 (2.4)<br>14 (5.6)	1 (1.1)<br>4 (4.3)	3 (2.2)<br>9 (6.7)		1 (1.1)<br>3 (3.4)	3 (2.2)<br>11 (8.1)	
Median CFR in assessed artery:<br>LAD<br>Cx<br>Dg<br>Mg<br>RCA	 <br>2.3 [1.6; 3.5]<br>2.2 [1.5; 3.4]<br>2.4 [1.9; 2.9]<br>2 [1.1; 3.8]<br>2.4 [1.9; 3.1]	 <br>1.4 [1.1; 1.8]<br>1.4 [0.9; 1.7]<br>-<br>1.1 [0.9; 1.2]<br>1.1 [0.8; 1.6]	 <br>3.2 [2.5; 4.3]<br>3.3 [2.6; 3.6]<br>2.6 [2.1; 3.1]<br>3.8 [2.8; 4.7]<br>3.1 [2.4; 4.1]	<0.001	 <br>2 [1.4; 2.5]<br>1.8 [0.8; 2.8]<br>-<br>-<br>2.4 [0.7; 2.4]	 <br>3.0 [1.9; 4.1]<br>2.5 [1.7; 3.4]<br>2.6 [2.1; 3.1]<br>2.8 [1.2; 4.7]<br>3.1 [1.9; 4.1]	 <br><0.001<br>0.20<br>-<br>-<br>0.27
Median IMR in assessed artery:<br>LAD<br>Cx<br>Dg<br>Mg	 <br>20 [13; 34]<br>19 [13; 30]<br>17 [11.5; 22.5]<br>13.5 [9.0; 95.5]	 <br>28 [16; 45]<br>31.5 [13; 36]<br>-<br>95.5 [16; 175]	 <br>17 [12; 27]<br>14 [11; 24]<br>17 [6; 17]<br>9 [7; 11]	 <br><0.001<br>0.08<br>-<br>0.25	 <br>37 [29; 49]<br>33 [28; 41]<br>-<br>-	 <br>14 [11; 18]<br>13 [11; 15]<br>17 [6; 17]<br>11 [7; 16]	<0.001

RCA	21 [18; 32]	32 [17.5; 41]	20 [18; 22]	0.32	32 [32; 42]	20 [17; 22]	
Artery with abnormal CFR							
LAD	75 (81.5)				43 (82.7)	30 (78.9)	
Cx	10 (10.9)	-	-	-	6 (11.5)	4 (10.5)	0.63
Dg	1 (1.1)				1 (1.9)	-	
Mg	2 (2.2)				1 (1.9)	1 (2.6)	
RCA	4 (4.3)				1 (1.9)	3 (7.9)	
Artery with abnormal IMR							
LAD	74 (85.1)	43 (82.7)	31 (88.6)				
Cx	8 (9.2)	6 (11.5)	2 (5.7)	0.55	-	-	-
Dg	1 (1.1)	1 (1.9)	0 (0.0)				
Mg	1 (1.1)	1 (1.9)	0 (0.0)				
RCA	3 (3.4)	1 (1.9)	2 (5.7)				

Data are presented as median [Q1; Q3] and counts (percentages).

Abbreviations: see Table 2. FFR: fractional flow reserve; RFR: resting full-cycle ratio

Table S5. Coronary microcirculatory assessment with regard to FFR.

Variable	Total N = 249 (arteries)	High FFR (>0.8)		*P*-value	Low FFR (≤0.8)		*P*-value
		CMD N = 109 (arteries)	nonCMD N = 93 (arteries)		CMD N = 18 (arteries)	nonCMD N = 29 (arteries)	
RFR	0.92 [0.88; 0.95]	0.93 [0.91; 0.96]	0.92 [0.90; 0.95]	0.51	0.79 [0.71; 0.87]	0.85 [0.84; 0.88]	0.05
FFR	0.88 [0.83; 0.92]	0.90 [0,86; 0.93]	0.90 [0.86; 0.93]	0.73	0.73 [0.68; 0.78]	0.77 [0.72; 0.79]	0.31
CFR	2.3 [1.6; 3.4]	1.7 [1.2; 2.3]	3.3 [2.6; 4.7]	<0.001	1.55 [1.20; 1.90]	3.05 [2.8; 4.2]	<0.001
IMR	20 [13; 33]	32 [25; 44]	15 [12; 19]	<0.001	18.5 [13; 25]	11 [9; 14]	0.003

Data are presented as medians [Q1; Q3].

CFR: coronary flow reserve; CMD: coronary microvascular dysfunction; FFR: fractional flow reserve; IMR: index of microcirculatory resistance; RFR: resting full-cycle ratio.

Table S6. Treatment after coronary microcirculatory assessment

Variable	Total N = 223	CMD N = 125	nonCMD treated with PCI N = 24	nonCMD not treated with PCI N = 74	*P*-value
PCI	47 (21.1)	23 (18.4)	24 (24.5)		0.27
Conservative treatment	197 (88.3)	111 (88.8)	86 (87.8)		0.81
Added pharmacotherapy					
ACEI	18 (8.1)	13 (10.4)	1 (4.2)	4 (5.4)	0.34
BB	12 (5.4)	7 (5.6)	3 (12.5)	2 (2.7)	0.18
Nitrate	12 (5.4)	5 (4.0)	2 (8.3)	5 (6.8)	0.56
Ranolazine	0 (0.0)	0 (0.0)	0 (0.0)	0 (0.0)	-
Ivabradine	1 (0.4)	1 (0.4)	0 (0.0)	0 (0.0)	0.67
Trimetazidine	35 (15.7)	29 (23.2)	1 (4.2)	5 (6.8)	0.002
DHP CCB	38 (15.3)	33 (26.4)	0 (0.0)	5 (6.8)	0.001

NDHP CCB	4 (1.6)	4 (3.2)	0 (0.0)	0 (0.0)	0.20

Data are presented as counts (percentages).

Abbreviations: see Table 3.

Table S7. Factors associated with abnormal coronary microcirculation based on IMR and CFR measures – multivariable analysis.

Variable	OR	95% CI	*P*-value
Factors associated with CFR ≤2			
Intermediate stenosis in Cx artery	0.146	0.028 – 0.775	0.02
Factors associated with IMR ≥25			
Length of hospitalization in days	1.147	1.015 – 1.295	0.03
Intermediate stenosis in LAD artery	0.229	0.062 – 0.849	0.03
Cardiac Non- invasive Diagnostic Testing	0.157	0.029 – 0.840	0.03
Factors associated with IMR ≥25 and CFR ≤2			
Severe valve disease	24.821	1.393 – 442.206	0.03
Hyperlipidaemia	0.197	0.056 – 0.685	0.01

CFR: coronary flow reserve; Cx: circumflex (artery); IMR: index of microcirculatory resistance; LAD: left anterior descending (artery)
